# Supplementary material for: Learning to stand with unexpected sensorimotor delays
Source: eLife. 2021 Aug 10;10:e65085. doi: 10.7554/eLife.65085 (PMC8480973; doi:10.7554/eLife.65085)
Supplement: Transparent reporting form [file elife-65085-transrepform1.pdf]

eLIFE
1st Floor
24 Hills Road
Cambridge CB2 1JP, UK
P 01223 855340
W elifesciences.org
T @elife

eLife's transparent reporting form

We encourage authors to provide detailed information *within their submission* to facilitate the interpretation and replication of experiments. Authors can upload supporting documentation to indicate the use of appropriate reporting guidelines for health-related research (see EQUATOR Network), life science research (see the BioSharing Information Resource), or the ARRIVE guidelines for reporting work involving animal research. Where applicable, authors should refer to any relevant reporting standards documents in this form.

If you have any questions, please consult our Journal Policies and/or contact us: editorial@elifesciences.org.

Sample-size estimation

- You should state whether an appropriate sample size was computed when the study was being designed
- You should state the statistical method of sample size computation and any required assumptions
- If no explicit power analysis was used, you should describe how you decided what sample (replicate) size (number) to use

Please outline where this information can be found within the submission (e.g., sections or figure legends), or explain why this information doesn't apply to your submission:

Our sample-size estimation for these experiments was based on previously published work (Day et al. 1993; Fitzpatrick & McCloskey, 1994; Fitzpatrick et al. 1994a; Fitzpatrick et al 1994b; Kuo 2005; Mian & Day, 2014) and published work from our own research group (Dakin et al. 2007; Dakin et al. al. 2010; Blouin et al. 2011; Luu et al. 2012; Dalton et al. 2014; Héroux et al. 2015; Forbes et al. 2016; Rasman et al. 2018).

For evaluating standing balance behavior across different sensorimotor conditions for standing balance (all experiments), we and others have repeatedly demonstrated that 6 to 10 human participants provides sufficient power to discriminate standing behavior (e.g., variance of sway position and/or sway velocity) between experimental conditions (Day et al. 1993; Fitzpatrick et al. 1994b; Peterka 2002; Kuo 2005; Luu et al. 2011; Dalton et al. 2014; Héroux et al. 2015; Forbes et al. 2016; Rasman et al. 2018). We primarily evaluated standing balance behavior in Experiment 1 and 2. This led to our sample size for Experiment 1 (n = 13), Experiment 2 (n = 26) (16 of 26 completed the training protocol), and Experiment 3 (n = 7).

For vestibular-evoked muscle responses (Experiment 2 – vestibular testing & 3), we and others have demonstrated repeatedly that a sample of 5 to 10 human participants provides sufficient power to discriminate responses (coherence, gain and cross-covariance) between experimental conditions (Fitzpatrick et al. 1994a; Dakin et al. 2007; Dakin et al. al. 2010; Blouin et al. 2011; Luu et al. 2012; Mian & Day, 2014; Héroux et al. 2015; Forbes et al. 2016). Therefore, our sample size was 8 for Experiment 2 – vestibular testing and 7 for Experiment 3.

For perceptual detections and thresholds of standing self-motion (Experiment 2 – perceptual testing & 3), we and others have demonstrated that a sample of 5 to 10 human participants provides sufficient power to discriminate perceptual sensitivities between experimental conditions (Fitzpatrick & McCloskey, 1994; Luu et al. 2012). Therefore, our sample size was 18 for Experiment 2 – perceptual testing (10 participants in no-learning group, 8 in learning group) and 7 for Experiment 3.

The total number of participants can be found under the "Participants" heading in the Materials and Methods sections. The number of subjects in each experiment can be found in the first paragraph under each experiment heading in both the Results and the Material and Methods sections. In addition, references to our previous studies can be found in the current manuscript.

References

Blouin J-S, Dakin CJ, van den Doel K, Chua R, McFadyen BJ, Inglis JT. Extracting phase-dependent human vestibular reflexes during locomotion using both time and frequency correlation approaches. J Appl Physiol 111: 1484–1490, 2011.
Dakin CJ, Son GML, Inglis JT, Blouin J-S. Frequency response of human vestibular reflexes characterized by stochastic stimuli. J Physiol 583: 1117–1127, 2007.
Dakin CJ, Luu BL, van den Doel K, Inglis JT, Blouin J-S. Frequency-specific modulation of vestibular-evoked sway responses in humans. J Neurophysiol 103: 1048-1056, 2010.
Dakin CJ, Inglis JT, Chua R, Blouin J-S. Muscle-specific modulation of vestibular reflexes with increased locomotor velocity and cadence. J Neurophysiol 110: 86–94, 2013.
Dalton BH, Blouin J-S, Allen MD, Rice CL, Inglis JT. The altered vestibular-evoked myogenic and whole-body postural responses in old men during standing. Experimental Gerontology 60 : 120-8. 2014.
Day BL, Steiger MJ, Thompson PD, Marsden CD. Effect of vision and stance width on human body motion when standing : impliciations for afferent control of lateral sway. J Physiol 469 : 479-99. 1993.
Fitzpatrick RC, Burke D, Gandevia SC. Task-dependent reflex responses and movement illusions evoked by galvanic vestibular stimulation in standing humans. J Physiol 478 : 363-72. 1994a.
Fitzpatrick RC, Rogers DK, McCloskey DI. Stable human standing with lower-limb muscle afferents providing the only sensory input. J Physiol 480 : 395-403. 1994b.
Fitzpatrick RC, McCloskey DI. Proprioceptive, visual and vestibular thresholds for the perception of sway during standing in humans. J Physiol 478 : 173-86. 1994.
Forbes PA, Luu BL, Van der Loos HFM, Croft EA, Inglis JT, Blouin J-S. Transformation of vestibular signals for the control of standing in humans. J Neurosci 36: 11510–11520, 2016.
Héroux ME, Law TCY, Fitzpatrick RC, Blouin J-S. Cross-modal calibration of vestibular afference for human balance. PLOS One. 10: e0124532. 2015.
Kuo AD. An optimal state estimation model of sensory integration in human postural balance. Journal of neural engineering 2: S235-249, 2005.
Luu BL, Huryn TP, Van der Loos HF, Croft EA, Blouin J-S. Validation of a robotic balance system for investigations in the control of human standing balance. IEEE Engineering in Medicine and Biology Society 19: 382-390, 2011.
Luu BL, Inglis JT, Huryn TP, Van der Loos HF, Croft EA, Blouin J-S. Human standing is modified by an unconscious integration of congruent sensory and motor signals. J Physiol 590: 5783–94, 2012.
Peterka RJ. Sensorimotor integration in human postural control. J Neurophysiol 88: 1097-118. 2002.
Mian OS, and Day BL. Violation of the craniocentricity principle for vestibularly evoked balance responses under conditions of anisotropic stability. The J Neurosci 34: 7696-703, 2014.
Rasman BG, Forbes PA, Tisserand R, Blouin J-S. Sensorimotor manipulations of the balance control loop – beyond imposed external perturbations. Frontiers in neurology : 899, 2018.

Replicates

- You should report how often each experiment was performed
- You should include a definition of biological versus technical replication
- The data obtained should be provided and sufficient information should be provided to indicate the number of independent biological and/or technical replicates
- If you encountered any outliers, you should describe how these were handled
- Criteria for exclusion/inclusion of data should be clearly stated
- High-throughput sequence data should be uploaded before submission, with a private link for reviewers provided (these are available from both GEO and ArrayExpress)

Please outline where this information can be found within the submission (e.g., sections or figure legends), or explain why this information doesn't apply to your submission:

We report the results of three experiments, each of which included a different number of participants. Experiment 1 had 13 participants, Experiment 2 had 26 participants (with 16 participating in the training procedure) and Experiment 3 had 7 participants. For all experiments, each participant performed the experiment once. Thus, Experiment 3 was repeated 7 times (once on each participant). This information is provided in the Material & Methods section.

Biological replicates are when the same type of organism is grown/treated under the same conditions. Here, we only tested human participants, and each participant only performed one of the three experiments. Therefore, we did not define biological replicates in our paper. We did our best to control variability in our sample with a small variability in age, height and weight and excluding participants with known neurological deficits. This information is provided in the Material & Methods section, under the header "Participants", lines 497-503.

A technical replicate is the same sample tested multiple times. Here, each participant only performed one of the three experiments, and an experiment was only conducted once for each participant. Therefore, we did not define any of our participants as technical replicates.

Trial data were excluded from certain analyses for the following reasons. In Experiment 1 and 2, we evaluated standing behavior by extracting body sway velocity variance over 2 seconds windows of standing balance, where participants did not reach the balancing limits on the robot (6° anterior, 3° posterior) for at least 2 seconds. For a given condition (e.g., standing with a 500 ms delay), if a participant's data did not balance for at least one 2-s period without exceeding the balance limits, we did not extract a sway velocity variance for that trial. This is stated in the Materials and Methods sections (l. 789-815) under the header "Balance behavior" of the manuscript. In Experiment 2 – perceptual testing and Experiment 3, participants were asked to report unexpected standing motion after the robotic balance simulation transitioned from baseline to an imposed delay and quantified the frequency of their perceptual detections and associated detection latency. If participants pressed and held the button switch (which was used to indicate a perception) prior to and then during transition (i.e., a false detection that crosses into a correct detection after the transition), the transition was removed from analysis. This is stated in the manuscript in the Materials and Methods section (l. 816-825) and the number of used trials/transitions is reported in the Results section in Table 3 (pg. 57), in the text (l. 294-295) and the Figure 6 caption (l. 1307-1312).

No DNA data sequencing was performed in our study.

Statistical reporting

- Statistical analysis methods should be described and justified
- Raw data should be presented in figures whenever informative to do so (typically when N per group is less than 10)
- For each experiment, you should identify the statistical tests used, exact values of N, definitions of center, methods of multiple test correction, and dispersion and precision measures (e.g., mean, median, SD, SEM, confidence intervals; and, for the major substantive results, a measure of effect size (e.g., Pearson's r, Cohen's d)
- Report exact p-values wherever possible alongside the summary statistics and 95% confidence intervals. These should be reported for all key questions and not only when the p-value is less than 0.05.

Please outline where this information can be found within the submission (e.g., sections or figure legends), or explain why this information doesn't apply to your submission:

We evaluated the data using parametric (if normally distributed) and non-parametric (if not normally distributed) statistical methods. In Experiment 1, we used linear mixed model analyses to assess changes in whole-body sway behavior across delays (one test for sway velocity variance, one test for percent time within the virtual balance limits) and decomposed main effects using Bonferroni corrected pairwise comparisons. In Experiment 2, we compared the differences in sway velocity variance from different time points (e.g., 93rd minute of training vs one minute at baseline standing) using paired t-tests. We used the same comparisons using the variable – percent time within the balance limits. In Experiment 2 – vestibular testing, vestibular-evoked muscle response amplitudes were not normally distributed, so we used a non-parametric analysis to assess the effects of delay and learning. We rank transformed the data and ran an ordinal logistic regression, a non-parametric test that accounts for repeated measures and missing data. We then decomposed the main and interaction effects using Bonferroni corrected Wilcoxon signed-rank tests. For sway velocity variance in Experiment 2 – vestibular testing, we used a linear mixed model to assess changes in whole-body sway behavior and decomposed effects using Bonferroni corrected pairwise comparisons. In Experiment 2 – perceptual testing, we tested the effect of learning on perception by comparing 70% perception thresholds using a linear mixed model. We then decomposed main effects using Bonferroni corrected pairwise comparisons. Additionally, we tested the effect of delays and learning on changes in sway velocity variance during the perception trials in Experiment 2 – perceptual testing using a linear mixed model. We then decomposed main effects using Bonferroni corrected pairwise comparisons. In Experiment 3, we only reported descriptive statistics. We used a coherence analysis to determine if EVS-EMG time-varying coherence was significant, i.e. larger than a 99% confidence interval through frequencies and time, estimated using the number of repetitions (group data: 489 repetitions). We then assessed EVS-EMG time-varying gain alongside coherence, since gain values are only reliable when coherence is significant.

Statistical analysis methods are described and justified in the Material & Methods section, in the header "Statistical Analysis" (l: 945-1005).

Raw data of representative participants from different experiments and conditions are presented in Figure 1, 2 and 3. Details about the different signals presented can be found in the captions for Figures 1 (p. 52), Figure 2 (p. 52) and Figure 3 (p. 53).

We carefully reported the number of participants or trials used for the computation of all results (including decomposition of statistical test) as well as for the means ± standard deviation (results in text, tables), means ± standard error of means (error bars in Figure 2B, 3B, 4C, 5B, 5C, 6 top row), or medians with 25 and 75% quartiles (Experiment 2 vestibular data, Figure 4B) or interquartile ranges (Experiment 2 vestibular data, Table 2). We also provide a statistical summary table with the results of the main tests (Table 1; p. 55).

All p-values have been reported in the manuscript. P-values are reported in the Results section within the text (l. 116 – 320) and Table 1 (p. 55).

(For large datasets, or papers with a very large number of statistical tests, you may upload a single table file with tests, Ns, etc., with reference to sections in the manuscript.)

Group allocation

- Indicate how samples were allocated into experimental groups (in the case of clinical studies, please specify allocation to treatment method); if randomization was used, please also state if restricted randomization was applied
- Indicate if masking was used during group allocation, data collection and/or data analysis

Please outline where this information can be found within the submission (e.g., sections or figure legends), or explain why this information doesn't apply to your submission:

All participants belong to one of three experimental groups (Experiment 1, Experiment 2, Experiment 3). During Experiment 1, participants performed standing balance trials with different delays presented in an ascending order (to prevent larger delays having a cross-over influence on smaller delays). This information is provided in the Materials and Methods section of the manuscript (l. 669-673) under the heading "Experiment 1". For the training protocol used in Experiment 2 (including participants from both vestibular and perceptual testing groups), the training procedure was identical across participants. Participants first performed a pre-learning vestibular (vestibular testing) or perception experiment (perceptual testing) and then completed a training protocol where they practiced balancing with a 400 ms delay over 5 days. On the final day, a post-learning testing session (identical to pre-learning) was performed. Three months later, participants returned for a retention testing session (identical to pre and post testing). This information is provided in the Materials and Methods section of the manuscript (l. 685-700) under the subheading "Training Procedure and Timeline". In the vestibular testing sessions for Experiment 2 (pre-learning, post-learning, retention), the order of the delays tested was presented in four subgroups with randomized delay conditions (l. 721-723). This information is provided in the Materials and Methods section of the manuscript (l. 700-723) under the heading "Vestibular testing". In the perception testing sessions for Experiment 2 (no-learning, pre-learning, post-learning and retention), participants performed trials where the robot transitioned to different delays. These delays were randomly presented in each trial. This information is provided in the Materials and Methods section of the manuscript (l. 724-759) under the heading "Perceptual testing". In Experiment 3, the robot was programed to transition between baseline (20 ms) and 200 ms delays while participants received vestibular stimulation and performed the perception task. Therefore, the procedure was the same for each participant. This information is provided in the Materials and Methods section of the manuscript (l. 760-784) under the heading "Experiment 3".

No masking was used during group allocation, data collection nor data analysis.

Additional data files ("source data")

- We encourage you to upload relevant additional data files, such as numerical data that are represented as a graph in a figure, or as a summary table
- Where provided, these should be in the most useful format, and they can be uploaded as "Source data" files linked to a main figure or table
- Include model definition files including the full list of parameters used
- Include code used for data analysis (e.g., R, MatLab)
- Avoid stating that data files are "available upon request"

Please indicate the figures or tables for which source data files have been provided:

eLife Sciences Publications, Ltd is a limited liability non-profit non-stock corporation incorporated in the State of Delaware, USA, with company number 5030732, and is registered in the UK with company number FC030576 and branch number BR015634 at the address 1st Floor, 24 Hills Road, Cambridge CB2 1JP | August 2014

eLIFE
1st Floor
24 Hills Road
Cambridge CB2 1JP, UK
P 01223 855340
W elifesciences.org
T @elife

We have created a dataverse link for the source files needed to generate the group result figures. This can be found at https://doi.org/10.5683/SP2/IKX9ML. Source files will be published and publicly available upon acceptance for publication.
